# Editorial: Decision-making under stress: the importance of cortico-limbic circuits

**DOI:** 10.3389/fnbeh.2015.00203

**Published:** 2015-08-18

**Authors:** Ruud van den Bos, Gert Flik

**Affiliations:** Department of Organismal Animal Physiology, Radboud University NijmegenNijmegen, Netherlands

**Keywords:** stress, decision-making, prefrontal cortex, subcortical areas, limbic structures, cortisol

In many domains of society, such as the military, the police force, financial businesses, health care, and sports, decisions have to be made under stressful conditions. Often, only a thin line separates success from failure. In the latter case this may lead to personal dismay and public or political upheaval. For instance, here, Yu (2015) discusses the neuropsychological mechanisms of choking under pressure, i.e., while victory is within reach the subject fails; a disturbing experience indeed, as those who have experienced this will confirm. In his contribution he alludes to the prefrontal-subcortical circuits underlying choking under pressure; circuits involved in decisions and sensitive to the effects of stress.

Optimal decision-making and performance hinges on an interconnected set of prefrontal-limbic-striatal networks (e.g., Gläscher et al., 2012; van den Bos et al., 2014). Recently, studies have begun to disentangle the temporal and reciprocal dynamics of these brain networks following stressful events (e.g., Hermans et al., 2014; Quaedflieg et al., 2015; van den Bos, 2015). The stress response is characterized by activation of the sympatho-adrenomedullary (SAM)-axis, leading to (fast) release of catecholamines, and the hypothalamus-pituitary-adrenal cortex (HPA)-axis, leading to (slow) release of corticosteroids (cortisol, corticosterone) having both (immediate) non-genomic, and (late) genomic effects (Hermans et al., 2014; Quaedflieg et al., 2015). Not all subjects produce the same levels of catecholamines and/or corticoids, associated with physiological and behavioral differences, captured under the umbrella of coping styles (Koolhaas et al., 1999).

Here, several papers address the stress-related effects on (different structures in) these brain networks. Koot et al. (2014) show how corticosterone-injections into the infralimbic cortex hamper task-progress in male rats in a rodent version of the Iowa Gambling Task, a widely used decision-making task in humans. Lewis et al. (2014) show that stress has an effect on reward magnitude in a Pavlovian monetary task, accompanied by changes in the ventral putamen, especially in subjects that produce high levels of cortisol. Ter Horst et al. (2014) show in mice that the mineralocorticoid receptor (MR) is important in males, but not in females, in the ability to discriminate between conspecifics, for instance important in territorial defense, which may be related to hippocampal mediated inhibitory control. Using an innovative social decision-making task, Smith et al. (2014) show an intricate relationship between amygdala gene-expression levels of, among others, *brain-derived nerve growth factor*, plasma corticosterone levels and coping styles in male rats. Related to this Lambert et al. (2014) show an interaction in male rats between (predisposed) coping styles and reward-based training-schedules in dealing with uncertainty in which the hippocampus and lateral habenula are involved. Ly et al. (2014) in their contribution study how in women emotions induced by viewing faces may bias instrumental action, showing a delicate interplay between innate responses, Pavlovian processes and instrumental action. The different brain networks alluded to above may underlie their behavioral observations.

At the behavioral level acute stress promotes “here-and-now oriented” over “future oriented” behavior, and “automatic, less energetic” over “more explicit, energy costly” behavior (e.g., Schwabe and Wolf, 2013; Vogel et al., 2015). While at first sight one could thus predict that in the social domain this leads to a shift to selfish, egoistic, over altruistic, helping behavior in acute stress conditions—indeed some studies suggest this to be the case in moral decision-making (Starcke et al., 2011; see van den Bos et al., 2013 for review)—this is not always true as reviewed by Buchanan and Preston (2014). In their review, they point out the complex relationship between stress and social behavior, i.e., whether stress promotes pro-social behavior or not depends on the interaction between stress-induced changes in systems evolved to promote helping behavior, the risks at stake for subjects when showing helping behavior, and cues emanating from distressed subjects.

The notion that many factors determine the outcome in the social domain, may be extended to stress-research in general, as indeed many factors affect the outcome of stress on laboratory tasks or in real-life situations. Some of these factors have already been indicated above, e.g., coping styles and sex differences. Other factors may relate to the time of performing a task or activity in relation to the stressor (Hermans et al., 2014; Quaedflieg et al., 2015), or even the task being used. Here, Pabst et al. (2013) show that the effects of stress are also dependent on how the task is framed using the Game of Dice Task: stress-related effects were found when the task was framed in the loss domain, but not in the gain domain. Furthermore, Faustino et al. (2015) in their review draw attention to the idea that appraisal is related to behavioral flexibility, and that individual biases for being optimistic or pessimistic may affect the outcome of the evaluation of new, potentially stressful, situations.

While most, if nearly not all, research on the effects of stress on emotion, cognition, and decision-making is conducted in mammals, we are happy to include one paper on zebrafish. Neo et al. (2015) show the behavioral effects of loud noise on fish behavior. The effects of acute and chronic stress on the behavior of zebrafish and underlying changes in brain networks will be a clear challenge for the future. While brains of zebrafish and mammals clearly differ in shape and gross neuro-anatomy, more and more studies show functionally similar structures in the (zebra)fish, such as the amygdala, hippocampus, and cortical areas (e.g., Broglio et al., 2005; Mueller, 2012; Aoki et al., 2013). Recent studies have already explored the effects of chronic stress or long-term elevated levels of cortisol on zebrafish behavior, showing similarities from what is known in e.g., rodents (Piato et al., 2011; Manuel et al., 2014; Gorissen et al., 2015). Undoubtedly future studies will unravel the catecholamine-driven and corticosteroid-driven changes in brain networks in fish species.

Activation of the stress-system is an adaptive physiological response allowing the organism to deal with challenges, such as being able to respond immediately, meeting current and/or upcoming metabolic demands and being able to store relevant environmental information, framed in the context of allostasis (McEwen and Wingfield, 2003). Only when the stress-axis does not come to rest, and stress becomes chronic, stress-related disorders may develop (Koolhaas et al., 2011). Studies on the effects of stress on brain networks should and need to be complemented by studies on cognition-related and emotion-related behavior in humans and animals in the context of the ecological niche of organisms to understand in full the immediate and long-lasting effects of stressors on the behavior of the subject, be it in real life or in laboratory tasks (van den Bos et al., 2013). This in turn will reveal the adaptive value of the stress-related changes in brain and behavior as well as when responses become maladaptive. As indicated here and elsewhere research already has shown the many factors that need to be disentangled to understand this in full: from individual differences, including gender/sex-differences to timing, task-related differences and domain-related differences. The papers in this Research Topics offer building blocks to enhance understanding of stress-related changes in brain-behavior relationships in the field of decision-making.

## Conflict of interest statement

The authors declare that the research was conducted in the absence of any commercial or financial relationships that could be construed as a potential conflict of interest.
